# Practical approach to detection and surveillance of emerging highly resistant *Mycobacterium tuberculosis* Beijing 1071-32-cluster

**DOI:** 10.1038/s41598-021-00890-7

**Published:** 2021-11-01

**Authors:** Igor Mokrousov, Anna Vyazovaya, Viacheslav Sinkov, Alena Gerasimova, Panayotis Ioannidis, Weiwei Jiao, Polina Khromova, Dimitrios Papaventsis, Oksana Pasechnik, João Perdigão, Nalin Rastogi, Adong Shen, Yuriy Skiba, Natalia Solovieva, Philip Suffys, Silva Tafaj, Tatiana Umpeleva, Diana Vakhrusheva, Irina Yarusova, Svetlana Zhdanova, Viacheslav Zhuravlev, Oleg Ogarkov

**Affiliations:** 1 St. Petersburg Pasteur Institute, 14 Mira Street, St. Petersburg, Russia 197101; 2 Scientific Centre of the Family Health and Human Reproduction Problems, Irkutsk, Russia; 3 National Reference Laboratory for Mycobacteria, Sotiria Chest Diseases Hospital, Athens, Greece; 4 National Clinical Research Center for Respiratory Diseases, Beijing Key Laboratory of Pediatric Respiratory Infection Disease, Beijing Pediatric Research Institute, Beijing Children’s Hospital, Capital Medical University, National Center for Children’s Health, Beijing, China; 5 Omsk State Medical University, Omsk, Russia; 6 Research Institute for Medicines (iMed.ULisboa), Faculdade de Farmácia, Universidade de Lisboa, Lisbon, Portugal; 7 WHO Supranational TB Reference Laboratory, Unité de la Tuberculose et des Mycobactéries, Institut Pasteur de la Guadeloupe, Abymes, Guadeloupe, France; 8 Children’s Hospital Affiliated to Zhengzhou University, Henan Children’s Hospital, Zhengzhou Children’s Hospital, Zhengzhou, China; 9 Almaty Branch of National Center for Biotechnology in Central Reference Laboratory, Almaty, Kazakhstan; 10 St. Petersburg Research Institute of Phthisiopulmonology, St. Petersburg, Russia; 11 Fundação Oswaldo Cruz, Rio de Janeiro, Brazil; 12 University Hospital “Shefqet Ndroqi”, Tirana, Albania; 13 Ural Research Institute for Phthisiopulmonology, National Medical Research Center of Tuberculosis and Infectious Diseases of Ministry of Health of the Russian Federation, Yekaterinburg, Russia; 14 Clinical Tuberculosis Dispensary, Omsk, Russia

**Keywords:** Evolution, Genetics, Diseases

## Abstract

Ancient sublineage of the *Mycobacterium tuberculosis* Beijing genotype is endemic and prevalent in East Asia and rare in other world regions. While these strains are mainly drug susceptible, we recently identified a novel clonal group Beijing 1071-32 within this sublineage emerging in Siberia, Russia and present in other Russian regions. This cluster included only multi/extensive drug resistant (MDR/XDR) isolates. Based on the phylogenetic analysis of the available WGS data, we identified three synonymous SNPs in the genes *Rv0144*, *Rv0373c*, and *Rv0334* that were specific for the Beijing 1071-32-cluster and developed a real-time PCR assay for their detection. Analysis of the 2375 genetically diverse *M. tuberculosis* isolates collected between 1996 and 2020 in different locations (European and Asian parts of Russia, former Soviet Union countries, Albania, Greece, China, Vietnam, Japan and Brazil), confirmed 100% specificity and sensitivity of this real-time PCR assay. Moreover, the epidemiological importance of this strain and the newly developed screening assay is further stressed by the fact that all identified Beijing 1071-32 isolates were found to exhibit MDR genotypic profiles with concomitant resistance to additional first-line drugs due to a characteristic signature of six mutations in *rpoB450*, *rpoC485*, *katG315*, *katG335*, *rpsL43* and *embB497*. In conclusion, this study provides a set of three concordant SNPs for the detection and screening of Beijing 1071-32 isolates along with a validated real-time PCR assay easily deployable across multiple settings for the epidemiological tracking of this significant MDR cluster.

## Introduction

*Mycobacterium tuberculosis* was initially regarded as a highly homogeneous population, yet, recent data demonstrated that this species is more genetically and functionally diverse than previously appreciated with diverging clinical significance associated with some of its phylogenetic clades, genetic families, or clonal complexes^1^. Studies carried out in the previously unexplored geographical regions and reanalysis of the available *M. tuberculosis* strains were able to pinpoint clinically and/or epidemiologically significant genotypes emerging in different world regions. In-depth analysis of such genotypes allows for detection of their specific markers associated with pathobiologically significant features such as drug resistance, hypervirulence, or transmissibility.

**Figure 1:**
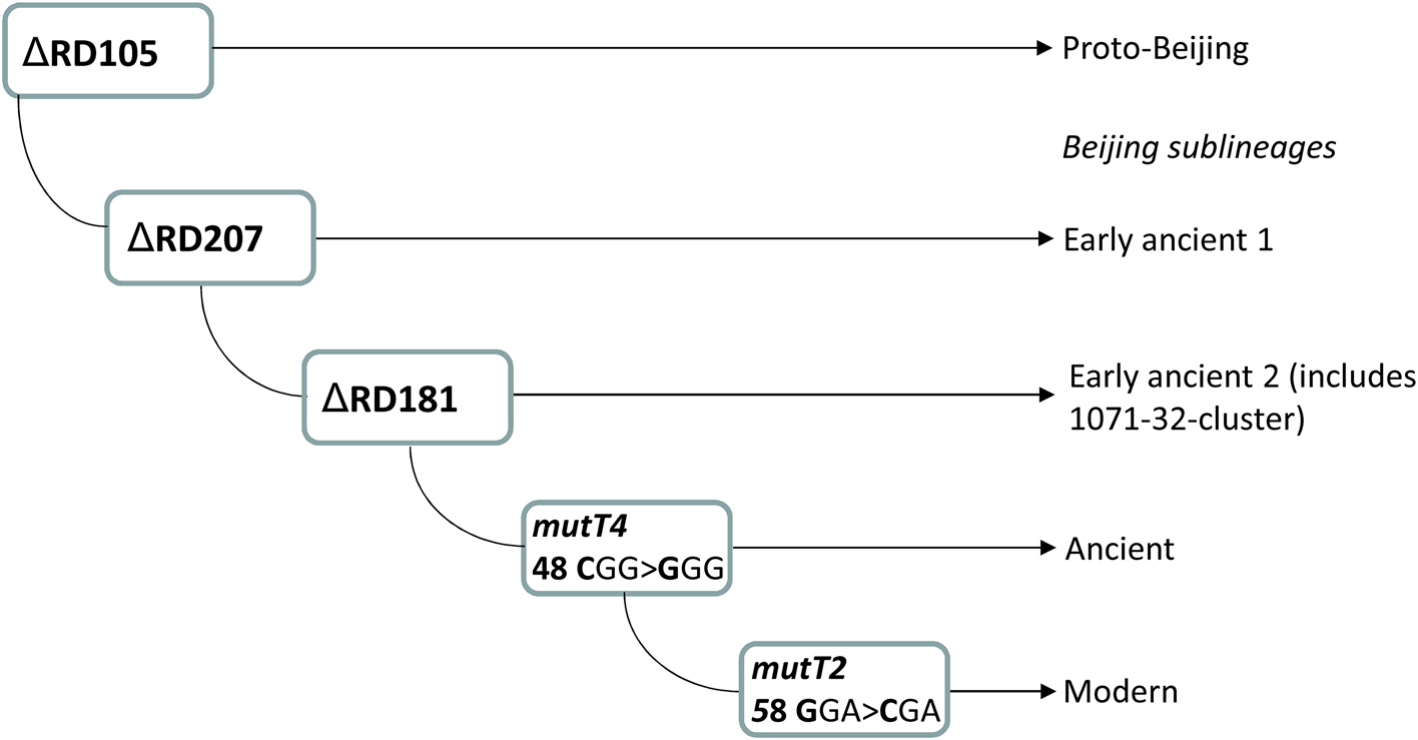
Simplified evolutionary pathway of the *M. tuberculosis* Beijing genotype. Digital profile of the 1071-32-cluster: 244231342644425173353923 (loci are listed in clockwise order on chromosome).

The Beijing genotype remains the most studied genetic family of *M. tuberculosis*. It can be robustly subdivided into phylogenetically ancient/ancestral and modern sublineages. Initially, subdivision was based on IS*6110*-RFLP typing, with ancient strains having classical spoligotypes and atypical IS*6110*-RFLP profiles^2^. Further studies had identified additional markers, mostly SNP and large sequence polymorphisms, that allowed some degree of discrimination within the ancient sublineage^3,4^ (Fig. 1). To date, WGS and high-resolution MIRU-VNTR analysis have proven useful for the identification of epidemic clusters but mostly within the globally dispersed modern Beijing sublineage^5–9^. In contrast, strains of the ancient Beijing sublineage circulate mainly in East Asia (K-strain in Korea is a known example^10^) and are rarely isolated elsewhere. Consequently, these strains are less studied, their epidemic impact is underestimated and their emergence in locations beyond East Asia is unexpected and needs further attention.

Modern Beijing strains are dominant in the former Soviet Union, the most notorious clusters being the Russian epidemic strain B0/W148^8^ and Central Asia Outbreak clade^9^. Both genotypes have been described in other countries and in some studies they were shown to circulate outside immigrant communities, highlighting their potential to spread to the autochthonous population^11–13^. Ancient Beijing strains in Russia were much less studied because they accounted for only 2% of the local *M. tuberculosis* populations^2^. Interestingly, a recent study in the Omsk province in Western Siberia detected a relatively elevated 10% prevalence of the ancient Beijing sublineage^14^. Most of those isolates belonged to the 1071-32-cluster (according to the MIRU-VNTRplus.org MLVA MtbC15-9 classification scheme) and also formed a monophyletic cluster within the WGS-based phylogenetic tree^15^. All Russian isolates of this cluster (from St. Petersburg, Omsk, and Samara) were multi-drug resistant (MDR) due to the specific signature of six mutations in *rpoB*, *rpoC*, *katG*, *rpsL* and *embB*. It was complemented in most isolates with additional mutations in *pncA*, *gyrA*, *rrs*, or *eis* thus leading to pre-XDR or XDR phenotype. Most of those mutations were high-confidence, high resistance-level mutations according to the WHO guidelines^16^.

WGS/NGS is becoming less expensive and more available and while NGS technologies are not always accessible especially in remote areas, this could be remedied by centralization of WGS services^17^. However, it is unrealistic to implement NGS for routine TB diagnostics and surveillance in the high burden countries such as Russia given a large size of the country and 50/100,000 incidence i.e., 72,000 new cases in 2019 (https://data.worldbank.org/indicator/SH.TBS.INCD?locations=RU). This highlights the importance and interest in the development of methods for detection of epidemic clusters, ideally less time-consuming than 24-loci VNTR typing but with an equivalent turn-around time and less expensive than WGS.

Here, we identified in silico the SNP markers specific for the Beijing 1071-32-cluster and developed a real-time PCR-based procedure to reliably detect this genotype. We evaluated the specificity of these SNPs on a geographically and genetically diverse collection of isolates from East Asia and the former Soviet Union in which the Beijing genotype is endemic or epidemic.

## Materials and methods

### Study collections

The study samples included *M. tuberculosis* DNA collected between 1996 and 2020 either convenience samples or from prospective or cross-sectional studies. Samples were not biased with regard to drug resistance, comorbidities or other patient related characteristics.

The study was approved by the Ethics Committees of the Research Institute of Phthisiopulmonology (protocol 31.2 of February 27, 2017) and St. Petersburg Pasteur Institute (protocol 41 of December 14, 2017).

All methods were performed in accordance with the relevant guidelines and regulations.

### Genotyping

DNA was extracted from cultured *M. tuberculosis* isolates using the CTAB-based method^18^ (Russian isolates from northwestern Russia and Omsk, isolates from Belarus, Kazakhstan, Japan, China, Vietnam, and Brazil), DNA-Sorb-B kit (Interlabservis, Russia) (Russian isolates from Irkutsk, Buryatia, Yakutia), «Express-tub» kit (Syntol, Russia) (Russian isolates from Yekaterinburg and Yamalo-Nenets), incubation at 98ºC and sonication for 15 min (isolates from Greece), three cycles of hot-freeze cell disruption (isolates from Albania), GenoLyse® kit (Hain Lifescience) (isolates from Estonia). One microliter of the DNA extracted using sonication, boiling method or commercial kits and 10–20 ng of DNA extracted using CTAB method was used for PCR.

Spoligotyping and 24 loci MIRU-VNTR typing were performed according to standard protocols^19,20^.

TB Profiler database (http://tbdr.lshtm.ac.uk/) was used for genotypic detection of drug resistance. MDR, pre-XDR and XDR phenotypes were defined according to the World Health Organization definitions: MDR are strains resistant to isoniazid and rifampicin; XDR—resistant to isoniazid, rifampicin, fluoroquinolone, and a second-line injectable agent; pre-XDR—resistant to isoniazid, rifampicin and either a fluoroquinolone or a second-line injectable agent.

### Bioinformatics and phylogenetic analysis

Phylogenetic analysis of the ancient Beijing strains based on the WGS data obtained in our laboratory or retrieved from SRA NCBI, was described previously^15^ and the list of the accession numbers of the analyzed genomes is provided in the same publication. In brief, short sequencing reads were mapped to the reference genome of *M. tuberculosis* H37Rv (NC_000962.3) by using the Burrows-Wheeler Aligner v. 0.7.12^21^. Single nucleotide polymorphism (SNP) calling was performed by SAMtools v.0.1.19^22^. Variable call format (vcf) files were annotated by BSATool v.0.1^23^. Graphical presentation of the trees was done using TreeGraph (http://treegraph.bioinfweb.info) and iTOL v3 (http://itol.embl.de). Obtained VCF files were used for analysis. Single nucleotide polymorphisms (SNPs) with quality scores ≥ 20 were used for phylogenetic analysis with IQTREE software (http://www.cibiv.at/software/iqtree) by using ModelFinder (http://www.iqtree.org/ModelFinder) with the GTR2 + FO + G4 substitution model and 1,000 rapid bootstrap replicates. SNPs located in repetitive genome regions and in PE and PPE genes were removed from the analysis due to possible misalignments.

To assess the significance of amino acid substitutions, we used 4 parameters: Tang U-index, Grantham D-distance, BLOSUM62 distance, and Dayhoff log odds. They were calculated using in-house scripts, in particular, the snpMiner2 program^24^.

### Real-time PCR

Three SNPs at genome positions 170505, 451510 and 398918 specific of the Beijing 1071-32 cluster were tested by real-time PCR assays that were run under the same cycling conditions. Oligonucleotide primers and FAM and HEX labeled probes are listed in Table 1.

**Table 1:** Oligonucleotides primers and labeled probes used for detection of three synonymous SNPs specific of the Beijing 1071-32-cluster.

Gene, codon	Position in gene	Position in genome	Primer, probe, and sequence
*Rv0144* 74GCC/GCT	222 C>T	170505 C>T	170505F 5′-CCAACGGTAGGTACCAAGC 170505R 5′-GCTTCCGAGTCTCATCTGCT 170505C wt 5′-[HEX]GTTCAATGTCGCTCACGGC[C-LNA]G[BHQ1] 170505 T mut 5′-[FAM]GTTCAATGTCGCTCACGGC[T-LNA]G[BHQ1]
*Rv0373c* 98CCG/CCA	294 G>A	451510 C>T	451510F 5′-CGCATCGATGTGACTGCC 451510R 5′-CACGGCTTGTACGTCGTTG 451510G wt 5′-[HEX]GCCTGGCTTGGATGCC[G-LNA]ACA[BHQ1] 451510A mut 5′-[FAM]GCCTGGCTTGGATGCC[A-LNA]ACA[BHQ1]
*Rv0334* 87GCG/GCA	261 G>A	398918 G>A	398918F 5′-GAGTGAACATCAGCTACGC 398918R 5′-GGCCGTAGAAGATGTTGTC 398918G wt 5′-[HEX]TCAGCCTGACGGTCTGGC[G-LNA]CA[BHQ1] 398918A mut 5′-[FAM]TCAGCCTGACGGTCTGGC[A-LNA]CA[BHQ1]

wt, wild type; mut, mutant; LNA, locked nucleic acid.

PCR mix (25 µl) contained 3 mM MgCl_2_, 1 unit of hot start *Taq* DNA polymerase, 200 μM each of dNTP, and primers (8 pmol each) and probes (4 pmol each). RotorGene6000 (Corbette Research) and CFX96 (Biorad) thermal cyclers were used with the following cycling conditions: 95 °C, 10 min; followed by 40 cycles of 95 °C, 30 s; 60 °C, 10 s, 72 °C, 20 s (signal detection at 60 °C at wavelength 510 nm for FAM and 555 nm for HEX). No difference was found between results obtained in different thermal cyclers.

### Detection of drug resistance mutations

Six drug resistance mutations were previously shown to be specific for Beijing 1071-32-cluster^15^: KatG Ser315Thr, KatG Ile335Val, RpoB Ser450Leu, RpoC Asp485Asn, EmbB Gln497Arg, RpsL Lys43Arg. To detect these mutations we used different methods, some of which were previously described, as follows.

Rifoligotyping reverse hybridization assay was used to detect mutations in the *rpoB* hot-spot region^25^. Other mutations were detected using allele-specific PCR or PCR–RFLP assays followed by 1.5% agarose gel electrophoresis (Table 2). All methods were initially tested and optimized using DNA samples of Beijing 1071-32 isolates with available WGS data and H37Rv as wild type control.

**Table 2:** Molecular assays used for detection of six drug resistance/compensatory mutations specific of the Beijing 1071-32-cluster.

Gene	Genome position	Method of detection	Primers and RE (in case of PCR–RFLP)	Reference
KatG 315 AGC/ACC	2155168	PCR–RFLP	Fw 5′-GCAGATGGGGCTGATCTACG Rev 5′-AACGGGTCCGGGATGGTG RE: MspI	^26^
KatG 335 ATC/GTC	2155109	PCR–RFLP	Fw 5′-GCAGATGGGGCTGATCTACG Rev 5′-AACGGGTCCGGGATGGTG RE: AvaII	This study
RpoB 450 TCG/TTG	761155	Rifoligotyping	Fw 5′-GTCGCCGCGATCAAGGA Rev 5'-[Biotin]ACGTCGCGGACCTCCA	^25^
RpoC 485 GAT/AAT	764822	Allele-specific PCR	Fw 5′-CTGGAGCTGTTCAAGCCGTTC RevWT 5′-CGATGACCTCTTCGAGCACAGC RevMut 5′-CGATGACCTCTTCGAGCACAGC	This study
EmbB 497 CAG/CGG	4248003	PCR–RFLP	Fw 5′-TGTGCAGCCCACCGGCCTGA Rev 5′-TGTGCAGCCCACCGGCCTGA RE: MspI	This study
RpsL L43 AAG/AGG	781687	PCR–RFLP	Fw 5'-CAGCCCGCAGCGTCGTGGTG Rev 5'-GCTGCGTGCCTGTTTGCGGTTCTT RE: MboII	This study

RE, restriction endonuclease.

Allele-specific PCR was used to specifically detect *rpoC* 485 GAT>AAT mutation; primers were selected using WASP online tool at https://bioinfo.biotec.or.th/WASP/home. Both allele-specific primers contained mismatch at − 1 position at 3′-end to increase instability during annealing and increase specificity. Two separate PCR reactions were performed for each strain at the same PCR conditions and using the same forward primer and allele-specific mutant primers. PCR products for both reactions are identical 130 bp and are subjected to gel-electrophoresis in adjacent lanes for each strain for easier comparison.

*katG315* AGC>ACC mutation was detected using PCR–RFLP assay as described^26^. This mutation creates an additional site for *MspI* and the main digestion bands have a size of 153 and 132 bp in the case of wild type and mutant alleles, respectively.

*katG335* ATC>GTC mutation was detected using PCR–RFLP assay. This mutation creates an additional site for *AvaII* and the main digestion products have sizes 378 and 285 bp in the case of wild type and mutant alleles, respectively.

*rpsL*43 AAG>AGG was detected using PCR–RFLP assay. This mutation occurs within the only *MboII* site in the PCR product in case of the wild type allele. The main *MboII* digestion products are 210 and 60 bp in case of wild type allele and 270 bp in case of mutation.

*embB*497 CAG>CGG was detected using PCR–RFLP assay. This mutation creates an additional site for *MspI* and the main digestion products have sizes 170 and 135 bp in the case of wild type and mutant alleles, respectively.

## Results

### Identification of Beijing 1071-32-cluster specific SNPs

Phylogenetic analysis of 184 WGS samples of ancient Beijing sublineages demonstrated that all isolates with Beijing 1071-32 VNTR profile were also grouped within a monophyletic branch on the WGS tree^15^ (Fig. S1). This branch included only Russian isolates (Omsk region in Siberia, St. Petersburg in Northwestern Russia, Samara in Central Russia). For the purposes of the present study, we reanalyzed the genome variation data underlying this tree and identified SNPs that were specific for this branch that we termed “Beijing 1071-32-cluster”, i.e. 1071-32 and its single/double locus variants (Fig. S2). In total, 121 SNPs were identified: 15 in intergenic regions and 106 in the coding sequences. These latter included 56 nonsynonymous (2 nonsense and 54 missense) and 39 synonymous mutations. In silico based assessment of the significance of the amino acid substitutions revealed three synonymous mutations in *Rv0144*, *Rv0373c*, and *Rv0334* with null significance score by all four tested parameters (Tang U-index, Grantham D-distance, BLOSUM62 distance, and Dayhoff log odds). We used these three SNPs for the development of real-time PCR assays (Table 1). The synonymous SNPs reflect a neutral evolution non-influenced by selection pressure and unlikely to independently occur in different and unrelated phylogenetic groups. Moreover, *Rv0334* codes for a thymidylyltransferase from the lipopolysaccharide biosynthetic pathway deemed essential by analysis of saturated Himar1 transposon libraries^27^ and therefore unlikely to be affected by genomic deletion events.

We evaluated in silico the specificity of these SNPs by search in the *M. tuberculosis* genome diversity GMTV database^28^. They were not detected in the non-Beijing isolates and were detected only in 6 Beijing isolates from Samara, Central Russia included in the above WGS analysis i.e. isolates of this Beijing 1071-32-cluster itself.

### Real-time PCR: optimization, validation and screening

**Figure 2:**
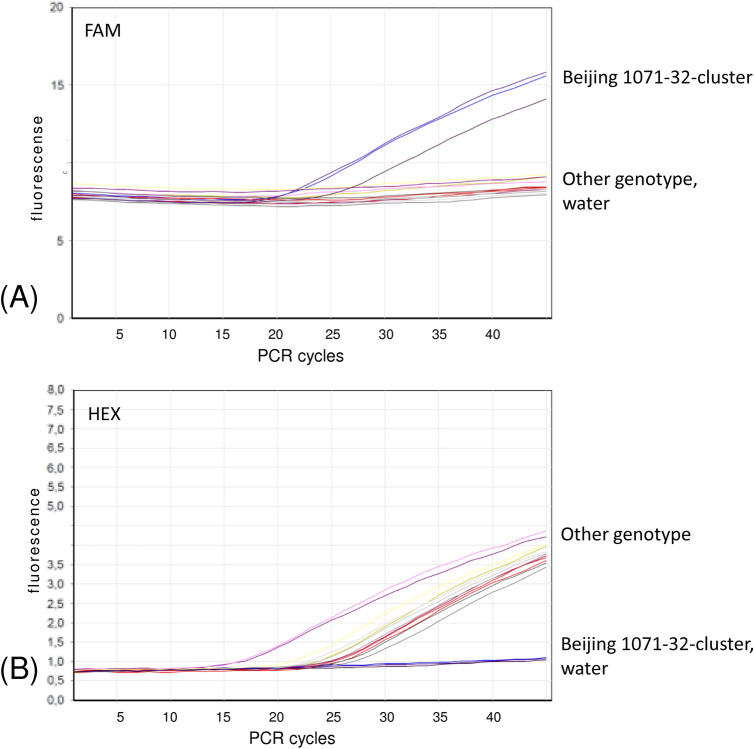
Fluorescence curves of a real-time PCR assay targeting Rv0144 SNP. (**A**) Beijing 1071-32 specific signal (FAM channel, 510 nm); (**B**) other genotype-specific signal (HEX channel, 555 nm). Water serves as negative control sample.

Three SNPs were targeted by three separate real-time PCR reactions run simultaneously under the same cycling conditions. Initial optimization was performed with DNA samples with available WGS and VNTR data that represented different VNTR clusters of the Beijing genotype and H37Rv reference strain. Clear discrimination between wild type and mutant alleles was observed for all three tested SNPs (see an example for one SNP in Fig. 2). The DNA used for analysis was extracted from cultured bacteria using different methods but this did not affect the performance of the assay.

The three RT-PCR assays were further applied to the 675 Beijing genotype isolates that represented different Beijing sublineages and had different VNTR profiles. These validation collections included isolates from Russia (n = 378), Kazakhstan (n = 109), China (n = 45), Vietnam (n = 37), Japan (n = 71), Albania (n = 5), Greece (n = 19), Brazil (n = 11). This analysis demonstrated that all 48 Beijing 1071-32-cluster isolates had these three mutations. In contrast, all other 627 isolates with other VNTR profiles had wild type alleles in these genome positions. Thus, this method has 100% sensitivity and 100% specificity to detect Beijing 1071-32-cluster.

**Figure 3:**
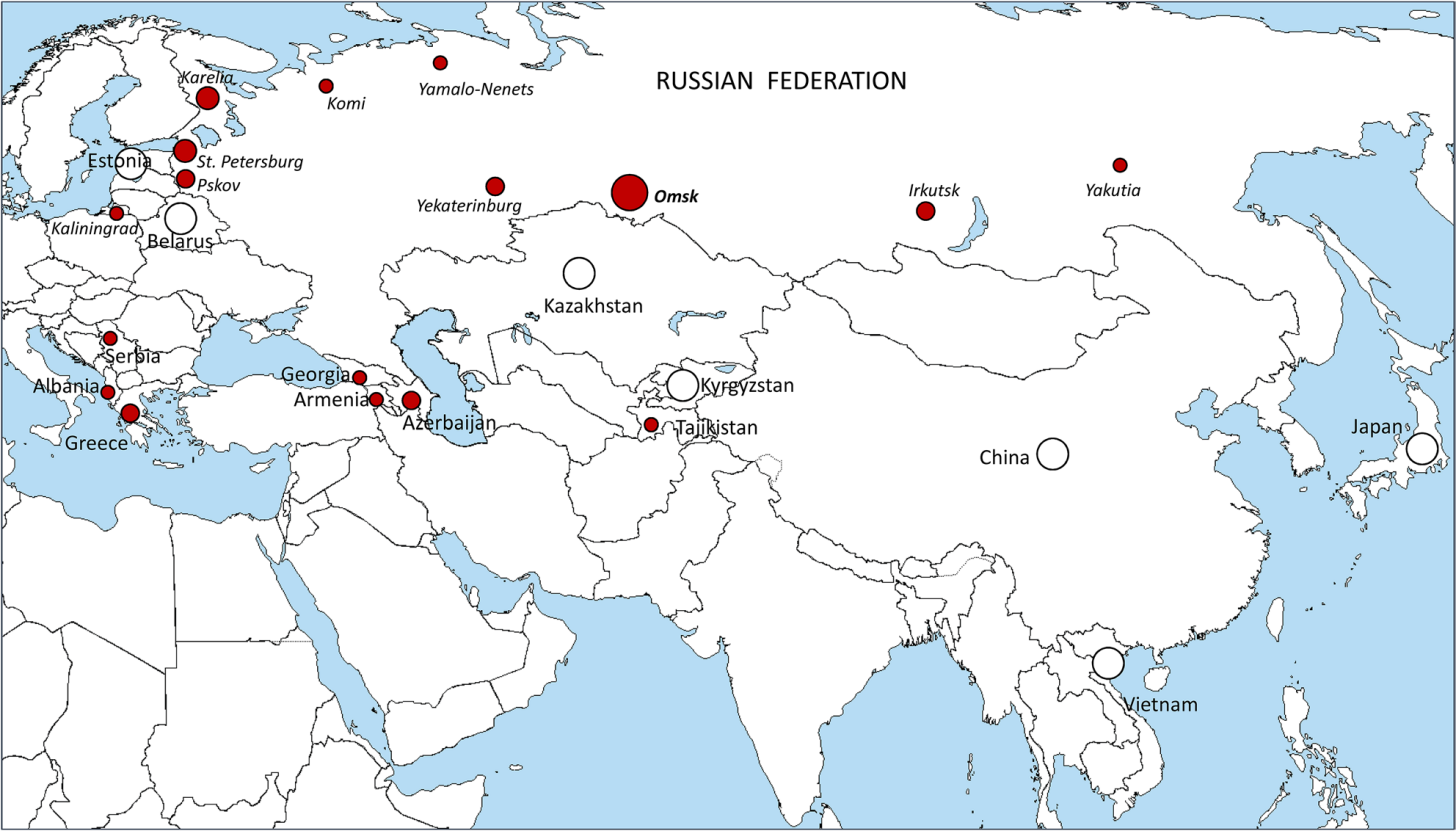
Geographic distribution of the Beijing 1071-32-cluster isolates identified in this study. Circle size roughly correlates with the proportion of identified isolates of this cluster (the smallest dots depict single isolates). Absence of these isolates in the local populations is shown by white circle. No Beijing 1071-312-cluster isolates were detected in Brazil (not shown on the map), Free map: https://commons.wikimedia.org/wiki/File:A_large_blank_world_map_with_oceans_marked_in_blue.PNG.

We further applied these RT-PCR assays to screen all available DNA collections from Russian regions and other countries. Results summarizing the above validation and screening analysis based on the total of 2375 isolates are shown in Table 3 and Fig. 3.

**Table 3:** Detection of the Beijing 1071-32-cluster by the developed PCR assay in local collections.

Country, region, years	Beijing	Beijing 1071-32
Russia, Northwest, CПб 1996–2020	835	22
Russia, Northwest, Karelia, 2014	43	6
Russia, Northwest, Pskov, 2015, 2018	85	3
Russia, Northwest, Kaliningrad, 2015	46	1
Russia, Northwest, Komi, 2017	73	1
Russia, Northwest, Vologda, 2018	51	0
Russia, Northwest, Murmansk, 2017	32	0
Russia. Siberia, Omsk, 2008–2019	372	37
Russia, Siberia, Irkutsk, 2014–2015	147	2
Russia, Ural, Yamalo-Nenets, 2017	101	1
Russia, Ural, Yekaterinburg, 2019–2020	85	2
Russia, Far East, Yakutia, 2012	67	1
Albania, 2007–2011	5	1
Greece, 2008–2011	19	3
Estonia, 1999 and 2014	91	0
Belarus, 2004	50	0
Kazakhstan, 2010	109	0
China, 2004	45	0
Vietnam, 2005	37	0
Japan, 2003–2005	71	0
Brazil, 2001–2007	11	0
Total	2375	80

Beijing genotype was detected using spoligotyping, or PCR targeting *dnaA*-*dnaN*::IS*6110* (Mokrousov et al., 2014) or based on VNTR-clustering. Beijing 1071-32-cluster was detected by real-time PCR analysis of SNPs in *Rv0144*, *Rv0373c*, and *Rv0334* as described in this study.

Geographic locations are shown on Fig. 3.

### Drug resistance mutational signature in the Beijing 1071-32-cluster

Under the previous WGS analysis, the isolates of the Beijing 1071-32-cluster were shown to have multiple mutations in drug resistance genes and, in particular, were characterized by the specific signature of 6 mutations *katG* 315AGC/ACC, *katG* 335ATC/GTC, *rpoB* 450TCG/TTG, *rpoC* 485GAT/AAT, *embB* 497CAG/CGG, *rpsL* 43AAG/AGG^15^.

We screened the above-identified Beijing 1071-32-cluster isolates for the presence of these mutations. As a result, all Beijing 1071-32-cluster isolates harbored these six mutations.

## Discussion

This study was undertaken to develop a practical approach to robust detection and prospective surveillance of the MDR/pre-XDR cluster within *M. tuberculosis* Beijing genotype emerging in Russia^14^. WGS-based analysis of the Russian and global datasets demonstrated that this Beijing 1071-32 cluster also presents a separate branch on the WGS-based tree^15^. In terms of large-scale sublineages, this cluster belongs to the early ancient Beijing sublineage characterized by deleted RD181 and wild type *mutT2*-58 and *mutT4*-48 codons (Fig. 1). In terms of drug resistance, it bears a characteristic signature of six drug resistance mutations associated with resistance to the four first-line drugs^15^. Additional mutations in different genes were also present in most isolates of that branch and were associated with resistance to one or more second-line drugs, leading to pre-XDR or XDR genotype (Fig. S2). Most noteworthy and alarmingly, this cluster included only MDR isolates. For example, even the notorious Russian epidemic B0/W148-cluster reportedly included not only MDR but polyresistant isolates resistant to STR and RIF or INH^8,29,30^.

On the whole, based on the VNTR analysis, Beijing 1071-32-cluster isolates were mostly detected in Russia with clearly increasing prevalence in Omsk, Siberia but these isolates were also present in other Russian regions and FSU countries in Central Asia and Transcaucasia (Fig. 3). In the large Beijing genotype VNTR dataset^31^, this VNTR profile 1071-32 was identified in the isolates from Armenia (1 isolate) and Abkhazia (5 isolates). When we additionally searched for the three neutral SNPs and six resistance mutations in the large Beijing genotype WGS dataset previously compiled and described in Perdigão et al.^13^, 5 more isolates that harbored these mutations were detected. They originated from the FSU countries Azerbaijan (n = 3), Georgia (n = 1) and Tajikistan (n = 1).

Outside FSU, the Beijing 1071-32 isolates were described in Serbia^31^, Albania^32^ and Greece^33^. These are Balkan countries but whether this fact reflects an already local circulation of this strain rather than independent importations from FSU is presently unknown. DNA from Albania and Greece were available for this study and we confirmed that they harbored all 9 targeted mutations i.e., 3 neutral SNPs and 6 resistance mutations. The three isolates from Greece were one from an immigrant from Georgia (FSU) and the remainder two from local Greek patients^33^. Moreover, Beijing genotype is extremely rare in Albania^32^ and it was previously speculated that these isolates in Albania could be linked to neighboring Greece. A large European multicenter study of drug resistant TB in the EU ten years ago identified the Beijing 1071-32 isolates and designated them as European resistant cluster ECDC_10^34^. This additionally highlights the presence of Beijing 1071-32 MDR strains in the EU and, given its association with MDR-TB, the relevance of their tracing on a more global scale not limited to Russia and other FSU countries.

Re-analysis of the different typing data available in our laboratory revealed that VNTR 1071-32 profile was detected in Russian strains isolated in 1996–1999 and these strains had a characteristic IS*6110*-RFLP profile designated as P0 in the in-house database^29^ (Fig. S3). Although the former gold standard IS*6110*-RFLP typing is almost never used anymore, this knowledge may be helpful to trace these isolates in the historical collections with IS*6110*-RFLP profiles available, e.g. RIVM database (Bilthoven, The Netherlands) or PHRI database (New Jersey, USA), thereby providing backward compatibility with more modern typing methods and WGS. We have also noticed that all Beijing 1071-32 isolates had 1 copy in the MIRU10 locus. This marker may serve for preliminary detection of these isolates among those assigned to the ancient sublineage of the Beijing genotype.

A strategy to target a limited number of SNPs (at least two) was recommended and applied to identify specific strains or clones by PCR based assays^11,35–37^ which definitely increases the robustness of identification. Thus, application of the entire set of the three targeted SNPs can be seen as the most robust method to detect the Beijing 1071-32-cluster. Nonetheless, detection of particular clusters/genotypes based on use of a single marker is an acceptable and parsimonious approach, provided that such marker was proven specific and sensitive in the validation studies and this concerns both detection of the particular clusters and families and the development of the SNP-barcode system^6,9,38,39^. In this view, since analysis of the three SNPs showed completely concordant results, testing of any of them appears the most practical and time-saving approach to trace this clinically significant MDR Beijing 1071-32-cluster.

Enigmatically, no fully susceptible or mono/polyresistant strains of this MDR cluster were identified yet, and circumstances of its origin remain unknown. Given that only 13 isolates with NGS data from three locations in Russia were analyzed in our previous paper^15^, we could not rule out that isolates with partial resistance profile could be identified in the large and geographically diverse collection in this study. However, no such isolates were detected and the fact that all geographically diverse isolates of this cluster bear the same 6–mutation drug resistance signature is remarkable. This variant included only MDR isolates and this finding favors the hypothesis of dissemination exclusively driven by primary resistance, rather than independent acquisition of resistances.

A Russian origin of this cluster is the most plausible scenario since it includes isolates from remote parts of Russia and some of the analyzed local collections in this study date back to 1996 (St. Petersburg). All identified strains of Beijing 1071-32, regardless of their origin, had all 6 resistance mutations. Given the geographic diversity of the isolates, this probably reflects the distant time of origin of this resistance mutation signature. Phylogenomic analysis suggested an origin of this Russian resistant cluster in the 1970s^15^ when RIF was first included in anti-TB chemotherapy^40^. This scenario finds parallel situations across Portugal and South Africa where M/XDR-TB endemic strains of the LAM lineage (e.g. Lisboa3 or KZN, respectively) are predicted to evolve to MDR following the introduction of RIF^41,42^.

The assay showed a good performance with diluted DNA extracted using simplified boiling procedure and we believe it may also work with DNA extracted from clinical samples using commercial kits. Such rapid detection would be especially useful also to reliably predict the MDR pattern since all isolates of this cluster were shown to be quadruple resistant.

In conclusion, this study provides a set of three concordant SNPs for the detection and screening of Beijing 1071-32 isolates along with a validated real-time PCR assay easily deployable across multiple settings for the epidemiological tracking of this important MDR cluster. Application of the entire set of the three targeted SNPs can be seen as the most robust method to detect the Beijing 1071-32-cluster. However, since analysis of these three SNPs showed concordant results, testing of any of them appears the practical and time-saving approach to trace this clinically significant emerging cluster. This assay may be especially useful in high MDR-TB burden and/or resource-limited countries of the former Soviet Union and in many world regions that receive migration flows from the FSU countries.

## Supplementary Information

Supplementary Information.

## Data Availability

The data that support the findings of this study are available from the corresponding author, I.M., upon request.

## Abbreviations

MDR: Multidrug resistance
XDR: Extensive drug resistance
WGS: Whole genome sequencing
NGS: Next generation sequencing
SNP: Single nucleotide polymorphism
MIRU: Mycobacterial interspersed repetitive units
VNTR: Variable number of tandem repeats
