# Supplementary material for: Practical approach to detection and surveillance of emerging highly resistant Mycobacterium tuberculosis Beijing 1071-32-cluster
Source: Sci Rep. 2021 Nov 1;11:21392. doi: 10.1038/s41598-021-00890-7 (PMC8560753; doi:10.1038/s41598-021-00890-7)
Supplement: Supplementary file 1 — Supplementary Information. [file 41598_2021_890_MOESM1_ESM.pdf]

Practical approach to detection and surveillance of emerging highly resistant *Mycobacterium tuberculosis* Beijing 1071-32-cluster

Igor Mokrousov*, Anna Vyazovaya, Viacheslav Sinkov, Alena Gerasimova, Panayotis Ioannidis, Weiwei Jiao, Polina Khromova, Dimitrios Papaventsis, Oksana Pasechnik, João Perdigão, Nalin Rastogi, Adong Shen, Yuriy Skiba, Natalia Solovieva, Philip Suffys, Silva Tafaj, Tatiana Umpeleva, Diana Vakhrusheva, Irina Yarusova, Svetlana Zhdanova, Viacheslav Zhuravlev, Oleg Ogarkov

SUPPLEMENTARY MATERIAL

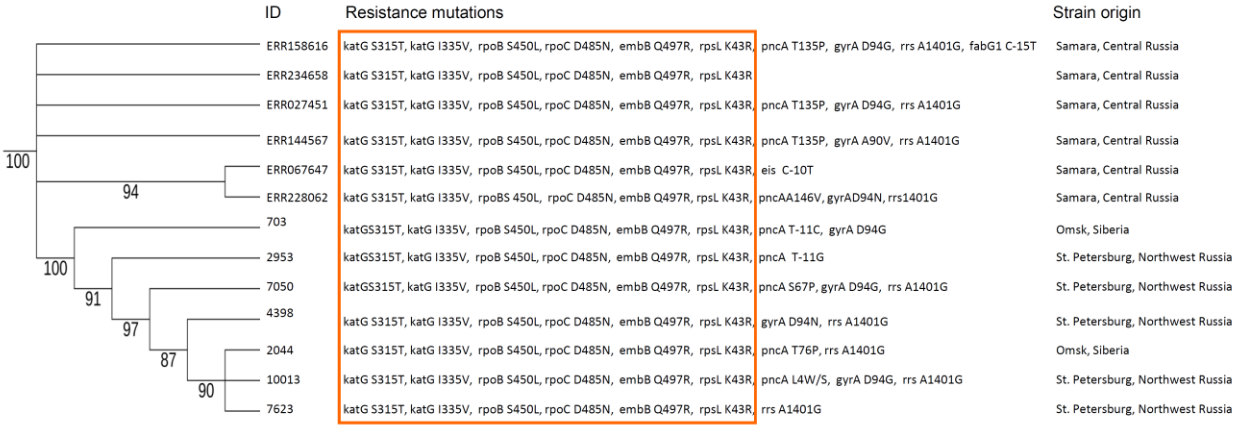

Figure S1. Branch of the WGS tree with cluster 1071-32 isolates.
Accession numbers: 4398 - SRR5266551, 7050 - SRR5266544, 7623 - SRR5266541, 2953 - SRR5266557, 10013 - SRR5266534, 2044 - SRR7796665, 703 - SRR7796666. Common resistance mutations are shown by orange box. Adapted from (Mokrousov et al., 2020).

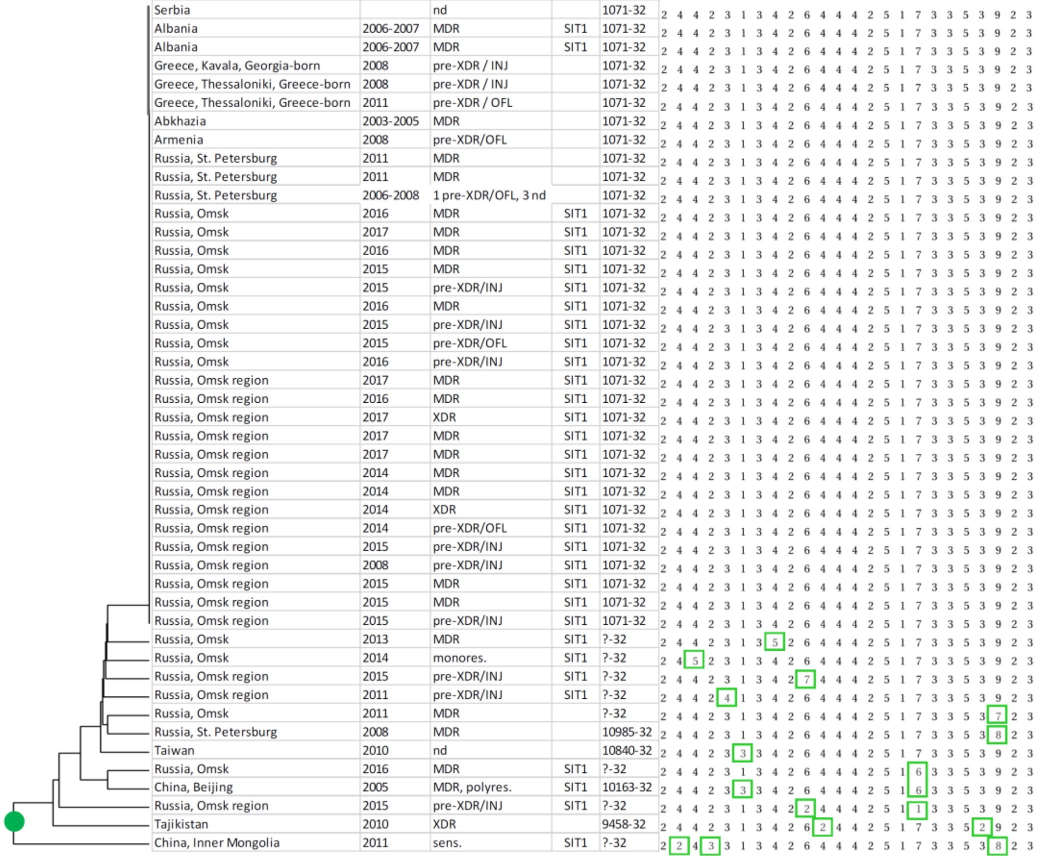

Country, region	Year	Resistance	SIT	MIva15-9	MIRU2	Mtub4	ETRC	MIRU4	MIRU40	MIRU10	MIRU16	Mtub21	MIRU20	QUB11b	ETRA	Mtub29	Mtub30	ETRB	MIRU23	MIRU24	MIRU26	MIRU27	Mtub34	MIRU31	Mtub39	QUB26	QUB4156	MIRU39
Serbia		nd		1071-32	2	4	4	2	3	1	3	4	2	6	4	4	4	2	5	1	7	3	3	5	3	9	2	3
Albania	2006-2007	MDR	SIT1	1071-32	2	4	4	2	3	1	3	4	2	6	4	4	4	2	5	1	7	3	3	5	3	9	2	3
Albania	2006-2007	MDR	SIT1	1071-32	2	4	4	2	3	1	3	4	2	6	4	4	4	2	5	1	7	3	3	5	3	9	2	3
Greece, Kavala, Georgia-born	2008	pre-XDR / INJ		1071-32	2	4	4	2	3	1	3	4	2	6	4	4	4	2	5	1	7	3	3	5	3	9	2	3
Greece, Thessaloniki, Greece-born	2008	pre-XDR / INJ		1071-32	2	4	4	2	3	1	3	4	2	6	4	4	4	2	5	1	7	3	3	5	3	9	2	3
Greece, Thessaloniki, Greece-born	2011	pre-XDR / OFL		1071-32	2	4	4	2	3	1	3	4	2	6	4	4	4	2	5	1	7	3	3	5	3	9	2	3
Abkhazia	2003-2005	MDR		1071-32	2	4	4	2	3	1	3	4	2	6	4	4	4	2	5	1	7	3	3	5	3	9	2	3
Armenia	2008	pre-XDR/OFL		1071-32	2	4	4	2	3	1	3	4	2	6	4	4	4	2	5	1	7	3	3	5	3	9	2	3
Russia, St. Petersburg	2011	MDR		1071-32	2	4	4	2	3	1	3	4	2	6	4	4	4	2	5	1	7	3	3	5	3	9	2	3
Russia, St. Petersburg	2011	MDR		1071-32	2	4	4	2	3	1	3	4	2	6	4	4	4	2	5	1	7	3	3	5	3	9	2	3
Russia, St. Petersburg	2006-2008	1 pre-XDR/OFL, 3 nd		1071-32	2	4	4	2	3	1	3	4	2	6	4	4	4	2	5	1	7	3	3	5	3	9	2	3
Russia, Omsk	2016	MDR	SIT1	1071-32	2	4	4	2	3	1	3	4	2	6	4	4	4	2	5	1	7	3	3	5	3	9	2	3
Russia, Omsk	2017	MDR	SIT1	1071-32	2	4	4	2	3	1	3	4	2	6	4	4	4	2	5	1	7	3	3	5	3	9	2	3
Russia, Omsk	2016	MDR	SIT1	1071-32	2	4	4	2	3	1	3	4	2	6	4	4	4	2	5	1	7	3	3	5	3	9	2	3
Russia, Omsk	2015	MDR	SIT1	1071-32	2	4	4	2	3	1	3	4	2	6	4	4	4	2	5	1	7	3	3	5	3	9	2	3
Russia, Omsk	2015	pre-XDR/INJ	SIT1	1071-32	2	4	4	2	3	1	3	4	2	6	4	4	4	2	5	1	7	3	3	5	3	9	2	3
Russia, Omsk	2016	MDR	SIT1	1071-32	2	4	4	2	3	1	3	4	2	6	4	4	4	2	5	1	7	3	3	5	3	9	2	3
Russia, Omsk	2015	pre-XDR/INJ	SIT1	1071-32	2	4	4	2	3	1	3	4	2	6	4	4	4	2	5	1	7	3	3	5	3	9	2	3
Russia, Omsk	2015	pre-XDR/OFL	SIT1	1071-32	2	4	4	2	3	1	3	4	2	6	4	4	4	2	5	1	7	3	3	5	3	9	2	3
Russia, Omsk	2016	pre-XDR/INJ	SIT1	1071-32	2	4	4	2	3	1	3	4	2	6	4	4	4	2	5	1	7	3	3	5	3	9	2	3
Russia, Omsk region	2017	MDR	SIT1	1071-32	2	4	4	2	3	1	3	4	2	6	4	4	4	2	5	1	7	3	3	5	3	9	2	3
Russia, Omsk region	2016	MDR	SIT1	1071-32	2	4	4	2	3	1	3	4	2	6	4	4	4	2	5	1	7	3	3	5	3	9	2	3
Russia, Omsk region	2017	XDR	SIT1	1071-32	2	4	4	2	3	1	3	4	2	6	4	4	4	2	5	1	7	3	3	5	3	9	2	3
Russia, Omsk region	2017	MDR	SIT1	1071-32	2	4	4	2	3	1	3	4	2	6	4	4	4	2	5	1	7	3	3	5	3	9	2	3
Russia, Omsk region	2017	MDR	SIT1	1071-32	2	4	4	2	3	1	3	4	2	6	4	4	4	2	5	1	7	3	3	5	3	9	2	3
Russia, Omsk region	2014	MDR	SIT1	1071-32	2	4	4	2	3	1	3	4	2	6	4	4	4	2	5	1	7	3	3	5	3	9	2	3
Russia, Omsk region	2014	MDR	SIT1	1071-32	2	4	4	2	3	1	3	4	2	6	4	4	4	2	5	1	7	3	3	5	3	9	2	3
Russia, Omsk region	2014	XDR	SIT1	1071-32	2	4	4	2	3	1	3	4	2	6	4	4	4	2	5	1	7	3	3	5	3	9	2	3
Russia, Omsk region	2014	pre-XDR/OFL	SIT1	1071-32	2	4	4	2	3	1	3	4	2	6	4	4	4	2	5	1	7	3	3	5	3	9	2	3
Russia, Omsk region	2015	pre-XDR/INJ	SIT1	1071-32	2	4	4	2	3	1	3	4	2	6	4	4	4	2	5	1	7	3	3	5	3	9	2	3
Russia, Omsk region	2008	pre-XDR/INJ	SIT1	1071-32	2	4	4	2	3	1	3	4	2	6	4	4	4	2	5	1	7	3	3	5	3	9	2	3
Russia, Omsk region	2015	MDR	SIT1	1071-32	2	4	4	2	3	1	3	4	2	6	4	4	4	2	5	1	7	3	3	5	3	9	2	3
Russia, Omsk region	2015	MDR	SIT1	1071-32	2	4	4	2	3	1	3	4	2	6	4	4	4	2	5	1	7	3	3	5	3	9	2	3
Russia, Omsk region	2015	pre-XDR/INJ	SIT1	1071-32	2	4	4	2	3	1	3	4	2	6	4	4	4	2	5	1	7	3	3	5	3	9	2	3
Russia, Omsk	2013	MDR	SIT1	?-32	2	4	4	2	3	1	3	5	2	6	4	4	4	2	5	1	7	3	3	5	3	9	2	3
Russia, Omsk	2014	monores.	SIT1	?-32	2	4	5	2	3	1	3	4	2	6	4	4	4	2	5	1	7	3	3	5	3	9	2	3
Russia, Omsk region	2015	pre-XDR/INJ	SIT1	?-32	2	4	4	2	3	1	3	4	2	7	4	4	4	2	5	1	7	3	3	5	3	9	2	3
Russia, Omsk region	2011	pre-XDR/INJ	SIT1	?-32	2	4	4	2	4	1	3	4	2	6	4	4	4	2	5	1	7	3	3	5	3	9	2	3
Russia, Omsk	2011	MDR		?-32	2	4	4	2	3	1	3	4	2	6	4	4	4	2	5	1	7	3	3	5	3	7	2	3
Russia, St. Petersburg	2008	MDR		10985-32	2	4	4	2	3	1	3	4	2	6	4	4	4	2	5	1	7	3	3	5	3	8	2	3
Taiwan	2010	nd		10840-32	2	4	4	2	3	3	3	4	2	6	4	4	4	2	5	1	7	3	3	5	3	9	2	3
Russia, Omsk	2016	MDR	SIT1	?-32	2	4	4	2	3	1	3	4	2	6	4	4	4	2	5	1	6	3	3	5	3	9	2	3
China, Beijing	2005	MDR, polyres.	SIT1	10163-32	2	4	4	2	3	3	3	4	2	6	4	4	4	2	5	1	6	3	3	5	3	9	2	3
Russia, Omsk region	2015	pre-XDR/INJ	SIT1	?-32	2	4	4	2	3	1	3	4	2	2	4	4	4	2	5	1	1	3	3	5	3	9	2	3
Tajikistan	2010	XDR		9458-32	2	4	4	2	3	1	3	4	2	6	2	4	4	2	5	1	7	3	3	5	2	9	2	3
China, Inner Mongolia	2011	sens.	SIT1	?-32	2	2	4	3	3	1	3	4	2	6	4	4	4	2	5	1	7	3	3	5	3	8	2	3

Figure S2. Branch of the VNTR tree with cluster 1071-32 (Mokrousov et al., 2019). Variant alleles (compared to 1071-32 profile) are shown by green boxes.

MIRU 2 154	Mtub 04 424	ETR C 577	ETR D (MIRU-4) 580	MIRU 40 802	MIRU 10 960	MIRU 16 1644	Mtub 21 1955	MIRU 20 2059	2163b QUB11b	ETR A 2165	Mtub 29 2347	Mtub 30 2401	ETR B 2461	MIRU 23 2531	MIRU 24 2687	MIRU 26 2996	MIRU 27 3007	Mtub 34 3171	MIRU 31 3192	Mtub 39 3690	QUB 26 4052	QUB-4156c 4156	MIRU39 4348
2	4	4	2	3	**1**	3	4	2	6	4	4	4	2	5	1	7	3	3	5	3	9	2	3

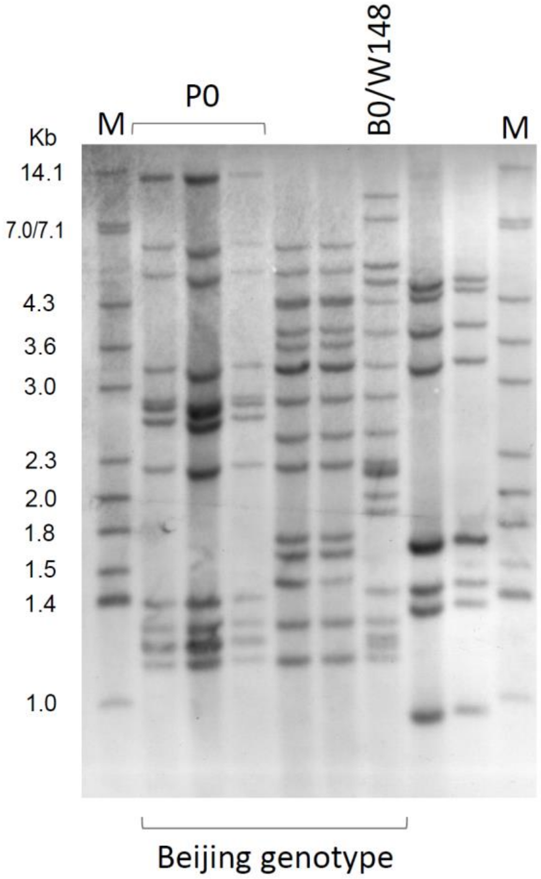

Figure S3. 24-MIRU-VNTR and IS*6110*-RFLP profile of the Beijing 1071-32-cluster.

On the IS*6110*-RFLP hybridization panel: M – molecular weight marker Mt14323/PvuII, P0 – characteristic profile of the Beijing 1071-32-cluster isolates, B0/W148 - Beijing B0/W148 isolate.
